# Effects of dietary glucosamine sulfate sodium on early laying performance and eggshell quality of laying hens

**DOI:** 10.1016/j.psj.2024.103982

**Published:** 2024-06-17

**Authors:** Ying Wang, Yanhua Huang, Panhong Zhou, Shengtao Lu, Jiale Lin, Guanglin Wen, Xiaoli Shi, Yuming Guo

**Affiliations:** ⁎ College of Animal Science and Technology, China Agriculture University, Beijing 100193, PR China; † College Animal Science, Guizhou University, Guiyang 550025, PR China; ‡ Key laboratory of Animal Genetics, Breeding and Reproduction in the Plateau Mountain Region, Ministry of Education, Guiyang 550025, PR China

**Keywords:** glucosamine, laying performance, eggshell quality, laying hen

## Abstract

This study was conducted to determine the influence of dietary glucosamine sulfate sodium (**GSS**) on laying performance, blood profiles, eggshell and inner quality of eggs and relative expression of the genes related to eggshell in laying hens at early stage. A total of 640 twenty-weeks-old Lohmann laying hens were randomly allotted to 4 treatments with 10 replicates of 16 hens each. The experiment lasted for 8 wk, and dietary treatments were: 1) CON, basal diet; 2) G1, CON + 0.2% GSS; 3) G2, CON + 0.4% GSS; 4) G3, CON + 0.6% GSS. The inclusion of GSS increased average daily feed intake, laying rate, and egg mass (*P* < 0.05) linearly during wk 21 to 25, 25 to 29, and 21 to 29, egg weight during wk 21 to 25 and 25 to 29, and improved (*P* < 0.05) feed conversion ratio linearly during wk 21 to 25. The supplementation of GSS increased (*P* < 0.05) albumen height quadratically, Haugh unit, calcium content, calcium mass, phosphorus content and phosphorus mass linearly at the end of 25th and 29th wk. At the end of 29th wk, the eggshell strength, eggshell weight, eggshell ratio, and eggshell thickness were increased (*P* < 0.05) linearly in GSS treatments compared with CON. The addition of GSS increased (*P* < 0.05) serum calcium, estrogen 2, and calcitonin, while decreased (*P* < 0.05) serum tartrate resistant acid phosphatase (**TRAP**), parathormone, IL-6 and prostaglandin E_2_ (**PGE_2_**) at the end of 29th wk. The inclusion of GSS increased (*P* < 0.05) the relative expression of ovocalyxin-32 and ovocalyxin-36 linearly at the end of 29th wk, and ovalbumin, osteopontin, calbindin 1, and ovocleidin-116 linearly at the end of 25th and 29th wk. Quadratic effects were observed (*P* < 0.05) in the laying rate during wk 21 to 25, serum TRAP and PGE_2_, the relative expression of ovocleidin-116 at the end of 29th wk. In summary, the inclusion of GSS up-regulated relative expression of osteopontin, ovocleidin-116, ovocalyxin-32 and ovocalyxin-36 in uterus, promoted the serum PGE_2_ and calcitonin, thus increased the calcium content of eggshell and finally enhanced eggshell quality.

## INTRODUCTION

If the health of pre-layer was poor, the laying hens would face a series of problems during the laying cycle, such as a decrease in laying rate, deterioration in shell quality and injury of ovarian health (Bain et al., 2016; Muir et al., 2022).

Glycosaminoglycans are linear, highly negatively charged polysaccharides consisting of repeated disaccharide units of hexosamine (glucosamine or galactosamine) and glyuronic acid (glucuronic acid or iduronic acid) or galactose (in keratoglycan sulfate). They have an anti-inflammatory action, minimize joint deterioration and prevent bone injury (Sgavioli et al., 2017). D-glucosamine sulfate is an amino sugar and a prominent precursor in the biochemical synthesis of glycosylated proteins and lipids, which is used as a dietary supplement. Glucosamine sulfate also is a natural constituent of glycosaminoglycans in the cartilage matrix and synovial fluid, which when administered exogenously, exerts pharmacological effects on osteoarthritic cartilage and chondrocytes (Bruyère et al., 2016). It is well documented that glucosamine sulfate could help the synovial fluid maintain greater resistance and elasticity (Toffoletto et al., 2005), inhibit chondroenzyme degradation (Wen et al., 2010), and further increase proteoglycan synthesis via chondrocytes (Henrotin et al., 2005). Furthermore, glucosamine sulfate is a substrate for the new synthesis of chondroitin sulfate as a precursor of glycosaminoglycans (Chan et al., 2006), which could help promote the synthesis of glycosaminoglycans or can reduce their degradation (Martins et al., 2023). Several studies showed that the supplementation of glucosamine sulfate alleviated leg conditions (Sgavioli et al., 2017), favored the development of the structure of the bones and joints (Santos et al., 2019; Martins et al., 2020; Martins et al., 2023) in broilers. These positive effects were also demonstrated in pigs (Wang et al., 2022), cattle (de Mattei et al., 2002), horses (Fenton et al., 2000), rats (Wolff, 2014), rabbits (Kamarul et al., 2011), dogs (Wenz et al., 2017), and in vitro (Kim et al., 2017).

The glycosaminoglycan could be extracted from the eggshell (Nakano et al., 2001). Moreover, a previous study indicated that glycosaminoglycans involved in maintaining the eggshell strength might influence the eggshell quality in laying hens (Liu et al., 2016). Accordingly, we hypothesized that dietary glucosamine sulfate might improve eggshell quality by reducing the use of bone calcium, promoting the absorption of calcium and increasing the bone density of laying hens. However, little information was available for glucosamine sulfate application in laying hens at early stage. Therefore, the objective of this study was to determine the effects of dietary glucosamine sulfate sodium (**GSS**) on laying performance, blood profiles, inner quality of eggs, eggshell quality and the relative expression of the genes related to eggshell in uterus of laying hens from 21 to 29 wk of age.

## MATERIALS AND METHODS

### Experimental Design and Hens Management

The animal feeding and management protocols for this study were approved by the Experimental Animal Ethics Committee of the Guizhou University (No: EAE-GZU-2021-T011). All efforts were made to minimize animal suffering.

A total of 640 Lohmann laying hens at 20 wk of age with similar body weight and uniform laying rate were selected in this 8-wk experiment. During the laying period, 4 hens were housed in a stainless-steel ladder cage (45  ×  45  ×  45 cm) with a replicate plot consisting of 16 hens in 4 adjoining cages. The cages were equipped with trough feeder and nipple drinker. All hens were housed in an environmentally controlled layer shed. The basic diet (Table 1) was corn–soybean meal based on NRC (1994) and Chinese Feeding Standard of Chickens (NY/T 33–2004). Dietary treatments included corn-soybean meal-based diet supplemented with 0, 0.2, 0.4, and 0.6% GSS. The D-glucosamine sulfate sodium product (99.5% purity, USP) was purchased from RIXING BIO-TEC Co. Ltd (Yangzhou, Jiangsu, China). The GSS was added at the expense of corn. Diets were fed in mash form. There were 10 replications per treatment and 16 hens per replicate in a randomized complete block design. The environmental temperature and humidity were kept at 24 ± 2°C and 65 ± 5%, respectively. A light schedule of 14L: 10D at an intensity of 15 lux. After 7 d acclimation, all hens were fed respective diets. Feed and water were provided ad libitum throughout this experiment.

**Table 1:** Basal diet composition (as-fed basis).

Ingredients	%
Corn	62.43
Soybean meal, CP 46%	19.30
Corn gluten meal, CP 60%	3.00
Wheat bran	3.00
Soybean oil	0.80
Coarse limestone	6.00
Fine limestone	3.13
Sodium bicarbonate	0.10
Sodium chloride	0.25
Choline chloride	0.10
L-Lysine·HCl	0.24
DL-Methionine	0.27
L-Threonine	0.03
Calcium hydrogen phosphate	0.75
Mineral and vitamin premix^1^	0.60
Chemical composition^2^	
ME, Kcal/kg^3^	2,650
Dry matter, %	88.94
Crude protein, %	15.70
Lysine, %	0.87
Methionine, %	0.54
Threonine, %	0.58
Tryptophan, %	0.16
Calcium, %	3.80
Total phosphorus, %	0.46
Calcium: total phosphorus	8.26

1 Premix provided the following kilogram of the diet: Vit A 9000 IU, Vit D_3_ 3000 IU, Vit E 35 mg, Vit K_3_ 1 mg, Vit B_1_ 2 mg, Vit B_2_ 6 mg, Vit B_6_ 4 mg, Vit B_12_ 0.02 mg, Niacin 35 mg, Folic acid 1 mg, Calcium pantothenate 20 mg, Biotin 0.25 mg, Choline Chloride 600mg; Fe (ferrous sulfate) 80 mg, Cu (copper sulfate) 10 mg, Mn (manganese sulfate) 100 mg, Zn (zinc sulfate) 90mg, I (calcium iodate) 1 mg, Se (sodium selenite) 0.3 mg.

2 Analyzed values.

3 Calculated values.

### Sampling and Measurements

The feed samples were ground to pass through a 1-mm sieve, and then analyzed for dry matter (method 934.01) and crude protein (N × 6.25; method 990.03) according to the American Society of Official Analytical Chemists (AOAC, 2002), as well as calcium and total phosphorus (985.01) according to the AOAC (2007). The amino acids of all diets were determined, following acid hydrolysis with 6 N HCl at 110°C for 24 h, using an amino acid analyzer (Biochrom 20, Pharmacia Biotech, Cambridge, England). Before acid hydrolysis, methionine and cystine were oxidized with formic acid. Tryptophan was determined after NaOH hydrolysis for 22 h at 110°C.

Egg production and egg weights were recorded daily, and feed intake was recorded weekly. These data were used to calculate the laying rate, average egg weight, average daily feed intake (**ADFI**), and feed conversion ratio at the stage of 21 to 25 and 25 to 29 wk. Laying rate (%) = total number of eggs laid during the statistical period / (number of housed hens × number of statistical days) × 100%. ADFI = total feed consumption during the statistical period / (number of housed hens × number of statistical days). Feed conversion ratio = total feed consumption during the test / total egg weight during the test. Egg mass production per hen per day was calculated as laying percentage multiplied by average egg weight of the hen. Average egg weight was determined per hen once a week.

At the end of 25th and 29th wk, 1 hen from each replicate was randomly selected and blood samples were collected by 5 mL tubes following the wing vein. The blood samples were then centrifuged at 3,000 × *g* for 10 min at 4°C and then serum was stored at -20°C till analysis. The serum calcium, tartrate resistant acid phosphatase (**TRACP**), estrogen 2 (**E_2_**), interleukin-6 (**IL-6**), prostaglandin E_2_ (**PGE_2_**), parathormone (**PTH**) and calcitonin (**CT**) levels were determined by Imark multifunctional automatic microplate reader (Bio-Rad, CA) following the instructions (Nanjing Jiancheng Bioengineering Institute, Nanjing, China).

Then, the same birds were sacrificed, and uterine tissues were sampled and snap frozen immediately in liquid nitrogen and stored at −80°C for further genes expression analysis. Premier 5.0 software was performed to design the primers according to the gene sequences of ovalbumin (**OVAL**), osteopontin (**OPN**), calbindin 1 (**CALB_1_**), ovocleidin-116 (**OC-116**), ovocalyxin-32 (**OCX-32**), ovocalyxin-36 (**OCX-36**) published in GeneBank, and ribosomal protein S2 (**RP-S2**) was used as the internal reference. The primers were synthesized by Invitrogen Biothech Co., Ltd. (Invitrogen Biothech, Shanghai, China). The primers information was listed in Table 2. Total RNA in the uterine tissues were isolated using Trizol Regent (Thermo Fisher Scientific, MA) and following the manufacturer's instructions. The purity and concentration of extracted RNA were tested by 2100 biological analyzer (Agilent Tech, CA), and RNA quality was evaluated with 1% (wt:vol) agarose gel electrophoresis. RNA was reverse-transcribed using Revert aid First Stand cDNA Synthesis Kit 1622 (Thermo fisher scientific, MA) according to the manufacturer's procedures. The size of all amplified products was confirmed by agarose gel electrophoresis at 1.2% (wt/vol), and imaged in Biosens SC710 System (Bio-rad, CA). Real time quantitative PCR for determining the gene expression was performed on the CFX96 RT- qPCR System with (Bio-Rad). The reaction protocol was as follows: pre-denaturation at 95°C for 5min; denaturation at 94°C for 45s, annealing at 60°C for 10s, extending at 72°C for min for 35 Cycles, ending at 72°C for 8min, kept at 4°C. The melting curve was automatically set by the machine (base temperature 65°C, rising 0.5-94°C every 5s). The measurements were performed in triplicate and the relative mRNA expression levels were normalized to RP-S2 by the 2^-△△Ct^.

**Table 2:** Primer sequences for RT-qPCR analysis.

Genes^1^	Primer sequence (5‘–3‘)	Accession no	Product size, bp	Tm, °C
OVAL	F: GCTATGGGCATTACTGACG	NM_205152.2	141	60
	R: TGCTGACCCTACCACCTCT			
OPN	F: GTCGTCACAGACTTCCCCACA	NM_204535.4	126	60
	R: GAGCTTGCTCGCCTTCACC			
CALB1	F: TGTAGTGCTCTGGGTTTC	NM_205513.1	106	60.5
	R: TTGTGAACTGTGCCTTTG			
OC-116	F: CTGGGAGTGAGGGTGGTAGCC	NM_204569.1	170	61
	R: GATGTATGCGAACTCATCTATGCC			
OCX-32	F: TCCAAGAAGAGGACCACAGATTTT	NM_204534.4	123	59
	R: CCAAGCCCCACAGTTGAAGAT			
OCX-36	F: GGGATTGGGATGCAGTTGGTC	NM_001030861.1	128	59
	R: CTTGTGCTTTTGATGTTGGAGGTAA			
RP-S2	F: CAAGGCGGAGGATAAAGAATGG	NM_001277164.1	106	60
	R: ATGGGAAGGGAGAAGAGGTAGAT			

1 OVAL, ovalbumin; OPN, osteopontin; CALB1, calbindin 1; OC-116, ovocleidin-116; OCX-32, ovocalyxin-32; OCX-36, ovocalyxin-36; RP-S2, ribosomal protein S2.

All eggs from laying hens at the end of 25th and 29th wk were collected for the assessment of egg quality parameters. The DET-6,000 egg quality tester (NABEL, Tokyo, Japan) was employed to measure both eggshell strength and egg weight. An automatic egg quality analyzer (Robotmation, Tokyo, Japan) was utilized to assess albumen height, yolk color, and Haugh unit. Yolk and eggshell were separated manually, and the shell membrane was carefully removed from the eggshell. The egg yolk was weighed. The egg yolk color was assessed using the Roche scale, ranging from 1 (lightest) to 15 (darkest). The shells were washed and air dried. The air-dried eggshell was weighed to calculate eggshell ratio. Eggshell thickness (average of upper end, lower end, and the middle) was measured with ETG-5200 eggshell thickness gauge (Robotmation, Tokyo, Japan). The contents of calcium and phosphorus of the eggshell were measured according to the AOAC (2007).

### Statistical Analysis

All data were analyzed using the Mixed procedures of SAS (SAS Inst. Inc., Cary, NC) with the replicate being the experimental unit. Orthogonal polynomials were used to assess the linear, quadratic and cubic effects of increasing dietary glucosamine sulfate sodium. Variability in the data is expressed as standard error of the means (**SEM**) and a probability level of *P* < 0.05 were considered to be statistically significant.

## RESULTS

### Laying Performance

The supplementation of GSS increased ADFI, laying rate and egg mass linearly (*P* < 0.05) during wk 21 to 25, 25 to 29 and 21 to 29 (Table 3). During wk 21 to 25 and 25 to 29, egg weight was increased linearly (*P* < 0.05) in GSS treatments compared with CON, while did not differ (*P* > 0.05) during wk 21 to 29. The inclusion of GSS improved (*P* < 0.05) feed conversion ratio linearly during wk 21 to 25, but did not affect (*P* > 0.05) feed conversion ratio during wk 25 to 29 and 21 to 29. There were no quadratic or cubic effects (*P* > 0.05) on ADFI, egg weight or feed conversion ratio except the laying rate for quadratic effect during wk 21 to 25.

**Table 3:** Effect of dietary GSS on laying performance in laying hens.^1^

Item^2^	CON	G1	G2	G3	SEM^3^	*P-*value^4^		
						Linear	Quadratic	Cubic
ADFI, g								
20 wk	107.46	106.98	107.25	107.11	0.103	0.510	0.631	0.476
21–25 wk	109.17	111.62	115.78	114.37	1.469	0.023	0.446	0.491
25–29 wk	112.97	113.18	116.51	115.87	0.910	0.013	0.833	0.376
21–29 wk	111.05	112.35	116.23	115.25	1.213	0.017	0.618	0.403
Laying rate, %								
20 wk	51.95	51.01	51.77	51.98	0.231	0.752	0.455	0.762
21–25 wk	79.22	79.83	81.76	85.00	1.301	0.026	0.001	0.684
25–29 wk	88.94	88.17	92.10	90.03	0.853	0.034	0.831	0.591
21–29 wk	84.08	84.00	86.93	87.52	0.927	0.042	0.826	0.539
Egg weight, g/egg								
20 wk	46.43	46.59	45.95	46.65	0.159	0.992	0.673	0.276
21–25 wk	53.93	54.70	54.18	54.96	0.236	0.046	0.995	0.760
25–29 wk	56.33	58.19	58.75	58.85	0.585	0.048	0.134	0.843
21–29 wk	55.13	55.95	55.97	56.41	0.267	0.067	0.613	0.840
Egg mass, g/hen								
20 wk	24.12	23.76	23.79	24.25	0.12	0.856	0.425	0.657
21–25 wk	42.72^b^	43.67^b^	44.30^b^	46.72^a^	0.85	0.039	0.157	0.843
25–29 wk	50.10^b^	51.31^b^	54.11^a^	52.98^b^	0.89	0.046	0.113	0.679
21–29 wk	46.35^b^	47.00^ab^	48.65^a^	49.37^a^	0.70	0.041	0.652	0.792
Feed conversion ratio								
20 wk	2.28	2.32	2.34	2.32	0.013	0.281	0.094	0.587
21–25 wk	2.09	2.02	2.08	2.02	0.019	0.041	0.943	0.064
25–29 wk	2.03	2.05	1.97	1.99	0.019	0.282	0.094	0.051
21–29 wk	2.13	2.13	2.13	2.11	0.005	0.487	0.943	0.764

1 Means represent 10 replicates per treatment of 16 hens per replicate.

2 ADFI: average daily feed intake.

3 Standard error of the means.

4 Linear, quadratic, and cubic effects of glucosamine sulfate sodium.

### Inner Quality of Eggs

The administration of GSS increased (*P* < 0.05) albumen height quadratically and Haugh unit linearly at the end of 25th and 29th wk (Table 4). No differences were observed (*P* > 0.05) in egg yolk weight, yolk index or yolk color throughout the experiment.

**Table 4:** Effect of dietary GSS on inner quality of eggs in laying hens.^1^

Item	CON	G1	G2	G3	SEM^2^	*P-*value^3^		
						Linear	Quadratic	Cubic
Albumen height, mm								
20 wk	7.77	7.99	7.89	7.90	0.045	0.586	0.472	0.403
25 wk	7.57	7.96	8.17	8.14	0.138	0.102	0.041	0.389
29 wk	7.49	8.01	8.23	8.22	0.173	0.103	0.038	0.403
Haugh unit								
20 wk	91.58	92.72	92.30	92.66	0.262	0.305	0.592	0.287
25 wk	87.31	89.33	90.21	90.94	0.785	0.032	0.210	0.874
29 wk	84.63	86.36	87.21	87.94	0.712	0.023	0.209	0.796
Egg yolk weight, g								
20 wk	9.79	9.93	9.84	9.80	0.031	0.879	0.387	0.439
25 wk	13.78	13.93	13.68	13.81	0.051	0.799	0.964	0.201
29 wk	13.96	13.80	13.97	13.98	0.042	0.652	0.580	0.332
Yolk index								
20 wk	21.93	21.10	21.39	21.44	0.172	0.558	0.385	0.401
25 wk	24.40	24.16	24.34	23.96	0.099	0.258	0.803	0.328
29 wk	24.40	24.16	24.34	23.96	0.099	0.258	0.853	0.319
Yolk color								
20 wk	12.68	12.17	12.45	12.81	0.140	0.692	0.223	0.363
25 wk	13.30	13.36	13.44	13.60	0.065	0.266	0.167	0.658
29 wk	13.56	13.43	13.81	13.89	0.107	0.174	0.666	0.547

1 Means represent 10 replicates per treatment of 16 eggs per replicate.

2 Standard error of the means.

3 Linear, quadratic, and cubic effects of glucosamine sulfate sodium.

### Eggshell Quality

The inclusion of GSS increased (*P* < 0.05) eggshell strength, eggshell weight, eggshell ratio and eggshell thickness linearly at the end of 29th wk (Table 5), while dietary treatments did not influence (*P* > 0.05) eggshell strength, eggshell weight, eggshell ratio or eggshell thickness at the end of 25th wk. At the end of 25th and 29th wk, the calcium content, calcium mass, phosphorus content and phosphorus mass were increased (*P* < 0.05) linearly in GSS compared with CON. The supplementation of GSS increased (*P* < 0.05) phosphorus content quadratically at the end of 25th and 29th wk.

**Table 5:** Effect of dietary GSS on eggshell quality in laying hens.^1^

Item	CON	G1	G2	G3	SEM^2^	*P-*value^3^		
						Linear	Quadratic	Cubic
Eggshell strength, kg/cm^2^								
20 wk	5.08	4.91	4.86	4.76	0.067	0.414	0.913	0.894
25 wk	5.21	5.06	4.92	4.94	0.067	0.125	0.753	0.376
29 wk	4.80	5.04	5.05	5.16	0.076	0.047	0.540	0.746
Eggshell mass, g/egg								
20 wk	5.26	5.04	5.11	4.98	0.060	0.174	0.521	0.735
25 wk	6.14	6.13	6.24	6.27	0.035	0.596	0.732	0.428
29 wk	6.50	6.57	6.82	6.87	0.091	0.037	0.925	0.894
Eggshell ratio, %								
20 wk	11.27	10.88	11.00	11.09	0.082	0.670	0.297	0.745
25 wk	11.14	11.42	11.74	11.62	0.131	0.134	0.314	0.453
29 wk	11.41	11.45	11.71	11.94	0.090	0.002	0.380	0.924
Eggshell thickness, mm								
20 wk	0.37	0.36	0.37	0.38	0.004	0.304	0.454	0.549
25 wk	0.39	0.38	0.39	0.38	0.002	0.778	0.836	0.386
29 wk	0.41	0.42	0.44	0.43	0.006	0.031	0.456	0.577
Calcium Content, %								
20 wk	34.55	34.71	34.43	34.67	0.063	0.600	0.883	0.379
25 wk	35.65	36.06	36.71	36.89	0.288	0.020	0.601	0.276
29 wk	36.14	36.14	36.75	36.98	0.214	0.049	0.695	0.496
Calcium mass, g/egg								
20 wk	1.81	1.75	1.76	1.72	0.018	0.308	0.706	0.681
25 wk	2.22	2.25	2.29	2.30	0.018	0.027	0.428	0.895
29 wk	2.22	2.26	2.29	2.31	0.019	0.014	0.339	0.912
Phosphorus content, %								
20 wk	0.17	0.17	0.17	0.17	0.001	0.225	0.268	0.764
25 wk	0.17	0.17	0.18	0.18	0.003	0.061	0.022	0.375
29 wk	0.17	0.17	0.18	0.19	0.004	0.041	0.041	0.287
Phosphorus mass, mg/egg								
20 wk	9.10	8.72	8.86	8.74	0.087	0.305	0.592	0.204
25 wk	10.55	10.71	10.98	11.46	0.199	0.033	0.230	0.931
29 wk	10.72	11.69	11.89	12.19	0.318	0.024	0.335	0.895

1 Means represent 10 replicates per treatment of 16 eggs per replicate.

2 Standard error of the means.

3 Linear, quadratic and cubic effects of glucosamine sulfate sodium.

### Blood Profiles

The administration of GSS increased (*P* < 0.05) serum calcium linearly at the end of 25th and 29th wk as well as serum E_2_ and CT at the end of 29th wk (Table 6). The inclusion of GSS reduced (*P* < 0.05) serum TRAP, PTH, and PGE_2_ at the end of 25th and 29th wk as well as serum IL-6 at the end of 29th wk. Quadratic effect was observed (*P* < 0.05) in serum TRAP and PGE_2_ at the end of 29th wk.

**Table 6:** Effect of dietary GSS on blood profiles in laying hen.^1^

Item^2^	CON	G1	G2	G3	SEM^3^	*P-*value^4^		
						Linear	Quadratic	Cubic
Calcium, mmol/L								
20 wk	1.37	1.38	1.37	1.36	0.004	0.316	0.268	0.216
25 wk	1.40	1.45	1.45	1.58	0.039	0.009	0.520	0.753
29 wk	1.38	1.43	1.55	1.54	0.042	0.010	0.624	0.658
TRAP, u/L								
20 wk	9.35	9.16	9.90	9.74	0.171	0.278	0.977	0.241
25 wk	10.71	10.18	9.13	8.48	0.503	0.012	0.819	0.907
29 wk	10.54	9.28	7.52	5.15	1.168	0.019	0.028	0.862
E_2_, ng/L								
20 wk	77.93	79.17	77.63	78.06	0.336	0.779	0.769	0.207
25 wk	73.55	76.70	76.68	79.61	1.237	0.053	0.949	0.847
29 wk	68.93	75.95	82.20	89.85	4.458	0.001	0.633	0.745
CT, ng/L								
20 wk	52.16	51.29	51.77	53.07	0.377	0.451	0.069	0.439
25 wk	51.27	52.12	52.29	55.06	0.823	0.094	0.415	0.843
29 wk	39.44	44.55	47.63	51.92	2.627	0.004	0.672	0.752
PTH, ng/L								
20 wk	109.89	112.01	113.85	110.07	0.931	0.835	0.245	0.485
25 wk	107.83	102.21	97.74	92.07	3.344	0.031	0.970	0.891
29 wk	104.67	101.65	98.41	86.79	3.911	0.022	0.255	0.904
IL-6, ng/L								
20 wk	53.20	56.33	52.85	51.61	1.004	0.470	0.469	0.387
25 wk	55.35	56.29	56.87	55.23	0.392	0.964	0.199	0.348
29 wk	61.94	50.24	46.80	39.73	4.636	0.001	0.544	0.869
PGE_2_, ng/L								
20 wk	91.00	92.51	92.41	91.04	0.416	0.997	0.094	0.420
25 wk	89.48	84.91	83.66	82.07	1.593	0.049	0.320	0.796
29 wk	74.41	71.89	63.54	47.96	5.967	0.036	0.030	0.833

1 Means represent 10 replicates per treatment of 1 hen per replicate.

2 TRACP, tartrate resistant acid phosphatase; E_2_, estrogen 2; CT, calcitonin; PTH, parathormone; IL-6, interleukin-6; PGE_2_, prostaglandin E_2_.

3 Standard error of the means.

4 Linear, quadratic and cubic effects of glucosamine sulfate sodium.

### The Relative Genes Expression Related to Eggshell

At the end of 25th and 29th wk, the inclusion of GSS up-regulated (*P* < 0.05) the relative expression of OVAL, OPN, CALB_1_ and OCX-116 linearly (Table 7). The supplementation of GSS increased (*P* < 0.05) the relative expression of OCX-32 and OCX-36 linearly as well as OCX-116 quadratically at the end of 29th wk.

**Table 7:** Effect of dietary GSS on relative genes expression related to eggshell in laying hens.^1^

Item^2^	CON	G1	G2	G3	SEM^3^	*P-*value^4^		
						Linear	Quadratic	Cubic
OVAL								
20 wk	1.02	1.06	1.04	1.01	0.011	0.709	0.197	0.679
25 wk	1.02	1.10	1.22	1.58	0.124	0.022	0.197	0.831
29 wk	1.00	1.10	1.32	1.58	0.129	0.017	0.140	0.903
OPN								
20 wk	1.02	1.05	1.08	1.03	0.013	0.707	0.268	0.680
25 wk	1.03	1.36	2.50	2.27	0.353	0.002	0.668	0.487
29 wk	1.08	1.30	2.48	2.40	0.364	0.010	0.813	0.742
CALB_1_								
20 wk	1.02	1.03	1.06	1.05	0.009	0.151	0.592	0.804
25 wk	1.01	1.53	2.87	2.92	0.481	0.001	0.706	0.932
29 wk	1.08	1.30	2.94	3.18	0.544	0.001	0.990	0.916
OCX-116								
20 wk	1.03	1.02	1.01	1.05	0.009	0.622	0.268	0.549
25 wk	1.01	1.07	1.32	1.38	0.091	0.037	0.989	0.870
29 wk	1.06	1.23	1.31	1.29	0.057	0.010	0.015	0.722
OCX-32								
20 wk	1.04	0.99	1.07	1.06	0.018	0.492	0.754	0.218
25 wk	1.01	1.25	1.23	1.33	0.068	0.114	0.541	0.301
29 wk	1.01	1.05	1.17	1.64	0.145	0.001	0.174	0.858
OCX-36								
20 wk	1.07	1.03	0.99	1.02	0.017	0.258	0.268	0.356
25 wk	1.03	0.98	1.25	1.17	0.062	0.284	0.936	0.204
29 wk	1.00	1.37	1.44	1.36	0.099	0.015	0.094	0.132

1 Means represent 10 replicates per treatment of 1 hen per replicate.

2 OVAL, ovalbumin; OPN, osteopontin; CALB_1_, calbindin 1; OCX-116, ovocleidin-116; OCX-32, ovocalyxin-32; OCX-36, ovocalyxin-36.

3 Standard error of the means.

4 Linear, quadratic and cubic effects of glucosamine sulfate sodium.

## DISCUSSION

### Laying Performance

Feed intake directly determines the amount of nutrient intake in poultry, and is closely related to the productive performance (Brickett et al., 2007). In the present study, the supplementation of 0.4% and 0.6% GSS increased ADFI by 4.66% and 3.78%, respectively, compared with CON. Similarly, feed intake was increased in rats after continuously injecting GSS at 150 nmol/min for 60 min by inhibiting glucokinase activity and then up-regulating the expression of hypothalamic neuropeptide Y and orexin (Osundiji et al., 2010; Zhou et al., 2011). In contrast, Sgavioli et al. (2017) reported that the addition of 0.12 to 0.24% glucosamine sulfate potassium did not influence ADFI in broilers. Similarly, dietary 0.15 to 0.3% glucosamine sulfate had no effects on ADFI in broilers (Martins et al., 2020). The above inconsistent results reflected that the effect of glucosamine sulfate on ADFI might be dose-dependent. The increase in ADFI resulted in the improvement in laying rate, egg weight and thus feed conversion ratio in the current study. Martins et al. (2020) observed that increasing levels of 0.15 to 0.3% glucosamine sulfate linearly increased weight gain in broilers. Notwithstanding, dietary 0.12 to 0.24% glucosamine sulfate did not affect body weight and weight gain in broilers due to the absence of positive effects on ADFI (Sgavioli et al., 2017). This discrepancy may be due to the species and dosage and more studies are needed to evaluate the effects of GSS on performance and its potential mechanism.

### Inner Quality of Eggs

Ovalbumin accounts for about 54% of the egg white and is the main ingredient in the egg white. Ovalbumin content and properties determine albumen height and Haugh unit, which reflect egg quality and freshness (Honkatukia et al., 2013). The acylated glucosamine sulfate is the glyco-based component of ovalbumin (Offengenden et al., 2011). Our study demonstrated that the inclusion of GSS increased albumen height quadratically and Haugh unit linearly, suggesting that GSS might promote ovalbumin formation. Ovalbumin decomposes, disulfide bond breaks, albumen height and egg freshness decreases (Stadelman et al., 1995). Moreover, the glucosamine sulfate can chelate Fe^2+^ well and scavenge oxygen free radicals and malondialdehyde (Yan et al., 2017), which indicated antioxidant properties to improve the protein secretion and Haugh unit.

### Eggshell Quality

It is well documented that both calcium and phosphorus content of eggshell critically influenced eggshell quality in laying hens (Summers, 1995; Dijkslag et al., 2021). In the current study, dietary GSS exerted a positive effect on the eggshell weight, eggshell thickness and eggshell strength at the end of 29th wk, which may be attributed to the increase in the calcium content, calcium mass, phosphorus content and phosphorus mass in laying hens. During the formation of eggshells, calcium determines their fragility, while phosphorus determines their toughness and elasticity. Similarly, dietary 0.03% glucosamine sulfate supplementation might increase the concentrations of calcium and phosphorus in the bone, thus benefiting the development of bird bones and cartilage in broilers (Santos et al., 2019).

### Blood Profiles

In the current study, the increased serum calcium may mirror the calcium content and mass in eggshell. Besides, the improvement in eggshell calcium content and eggshell quality may be due to the increased intestinal calcium absorption and the serum calcium in the present study. The serum calcium regulation is important for bone health and egg production. The three major organs that regulate blood calcium and calcium ion levels are intestine, bone and kidney (Seibel, 2005; Cransberg et al., 2001). The serum PTH, CT, E_2_ and cytokines (IL-6, TRAP) enhanced or inhibited dietary calcium absorption, bone calcium mobilization or kidney calcium reabsorption through these three organs to maintain serum calcium stability (Dijkslag et al., 2021). E_2_ plays an important role in maintaining the balance between bone resorption and bone formation (Chen et al., 2014). E_2_ limits bone turnover through osteocyte and osteoclast estrogen receptors. CT acts directly on osteoblasts, stimulating its proliferation and differentiation, and promoting chondrocyte formation, bone matrix synthesis and bone growth (Farley et al., 1991). TRAP is a specific marker of osteoclast differentiation and bone resorption (Seibel, 2005). The reduced serum TRAP indicated that GSS inhibited osteoclast differentiation and bone resorption in laying hens. Our previous study confirmed that dietary GSS down-regulated the relative expression of κβ receptor activating factor ligand, up-regulated osteoproteinogen, inhibited osteoclast differentiation, and thus decreased TRAP activity (Zhang, 2018). PTH is one of the most important hormones regulating calcium and phosphorus metabolism and bone turnover. It directly acts on bone cells and renal tubules, promotes bone calcium mobilization and renal calcium reabsorption, strengthens bone cells to dissolve bone calcium and osteoclasts to absorb bone matrix, and bone calcium can be continuously released to maintain blood calcium level (Zhang, 2018). Our study demonstrated that dietary GSS increased serum calcium, E_2_ and CT at the end of 29th wk, but decreased serum TRAP, PTH, IL-6, and PGE_2_. It is supposed that dietary GSS increased serum calcium by promoting dietary calcium absorption in intestinal tract, which was supported by Zhang (2018). It can be inferred that GSS does not increase serum calcium by enhancing bone mobilization, which is also supported by the reduced serum PTH by GSS addition. IL-6 is a bone resorption promoting factor that reflects the degree of bone loss and is regulated by E_2_ and PTH (Cheng et al., 2011). The results of this study showed that serum E_2_ was negatively correlated with serum IL-6, while serum IL-6 were positively correlated with PTH. The decreased IL-6 inhibited the activation of cyclooxygenase-2 and nitric oxide enzymes to induce the release of PGE_2_ and nitric oxide, increase their activities in poultry, promote inflammation, and lead to increased bone resorption (Cheng et al., 2011). Therefore, the inclusion of GSS promoted intestinal calcium absorption, increased serum calcium content, CT and E_2_, decreased serum PTH, IL-6 and PEG_2_, and thus reduced the utilization of bone calcium, increased the deposition of calcium and phosphorus in eggshell, and improved eggshell quality.

### The Relative Genes Expression Related to Eggshell

In the current study, the supplementation of GSS up-regulated the relative expression of OVAL, OPN, CALB_1_, OCX-32, OCX-36 and OCX-116 at the end of 29th wk, which may mirror the increased eggshell quality. OCX-116, involved in the agglutination and precipitation of calcium in uterine fluid (Hincke et al., 1999), regulates calcite growth during eggshell calcification, which affects eggshell elasticity, thickness, and shape. Osteopontin is adjacent to other gene sequences that regulates mineralization, forming a small integrin-binding ligand N-linked glycoprotein (**SIBLING**) locus. OCX-116 and other SIBLING proteins are members of the secretory calcium-binding phosphorylation protein (**SCPP**) family (Kawasaki and Weiss, 2006). Secretory calcium-binding phosphorylation protein family proteins generally bind calcium ions via acidic AA and phosphorylated serine (Kawasaki and Weiss, 2006). OPN is synthesized and secreted by eggshell glandular tubular epithelial cells and is an eggshell matrix protein that promotes eggshell calcification and eggshell formation. During eggshell calcification, OPN can bind to the growth surface of calcium carbonate crystals and affect the stability of crystal accumulation along the surface. OPN also participated in Ca^2+^ transport, and its expression level affected the deposition process of eggshell calcium carbonate crystals. The serum PTH may inhibit OPN expression, which was supported by our results. The up-regulation of OPN and OCX-116 gene expression indicated that GSS promoted the deposition of eggshell calcium. OCX-32, involved in the agglutination and precipitation of calcium in uterine fluid (Hincke et al., 1999), plays a role in the termination of eggshell calcification (Gautron et al., 1997), and the expression level of OCX-32 increases during the termination stage of eggshell formation (Dominguez-Vera et al., 2000). Dietary GSS up-regulated OCX-32 expression, which may prolong calcium deposition time, make calcium distribution more uniform, and improve eggshell quality. OCX-36 is mainly located in the eggshell near the shell membrane, which is highly conserved with lipopolypaccharide, etc., and has antibacterial and immune functions (Gautron et al., 2007). Dietary GSS increased the expression of OCX-36, indicating the ability of eggs to resist invasion by external microorganisms.

## CONCLUSIONS

The inclusion of 0.6% GSS increased ADFI and improved the serum calcium content, the calcium content of eggshell and the eggshell quality finally.

## DISCLOSURES

The authors declare no conflicts of interest.
